# Expectations of medical specialists about image-based teleconsultation – A qualitative study on acute burns in South Africa

**DOI:** 10.1371/journal.pone.0194278

**Published:** 2018-03-15

**Authors:** Lisa Blom, Lucie Laflamme, Helle Mölsted Alvesson

**Affiliations:** 1 Department of Public Health Sciences, Karolinska Institutet, Stockholm, Sweden; 2 University of South Africa, Pretoria, South Africa; University of California San Diego, UNITED STATES

## Abstract

**Background:**

Image-based teleconsultation between medical experts and healthcare staff at remote emergency centres can improve the diagnosis of conditions which are challenging to assess. One such condition is burns. Knowledge is scarce regarding how medical experts perceive the influence of such teleconsultation on their roles and relations to colleagues at point of care.

**Methods:**

In this qualitative study, semi-structured interviews were conducted with 15 medical experts to explore their expectations of a newly developed App for burns diagnostics and care prior to its implementation. Purposive sampling included male and female physicians at different stages of their career, employed at different referral hospitals and all potential future tele-experts in remote teleconsultation using the App. Positioning theory was used to analyse the data.

**Results:**

The experts are already facing changes in their diagnostic practices due to the informal use of open access applications like WhatsApp. Additional changes are expected when the new App is launched. Four positions of medical experts were identified in situations of diagnostic advice, two related to patient flow–clinical specialist and gatekeeper–and two to point of care staff–educator and mentor. The experts move flexibly between the positions during diagnostic practices with remote colleagues. A new position in relation to previous research on medical roles–the mentor–came to light in this setting. The App is expected to have an important educational impact, streamline the diagnostic process, improve both triage and referrals and be a more secure option for remote diagnosis compared to current practices. Verbal communication is however expected to remain important for certain situations, in particular those related to the mentor position.

**Conclusion:**

The quality and security of referrals are expected to be improved through the App but the medical experts see less potential for conveying moral support via the App during remote consultations. Experts’ reflections on remote consultations highlight the embedded social and cultural dimensions of implementing new technology.

## Introduction

Medical experts interact with other healthcare professionals in various ways and for different purposes [1–4]. In emergency care, experts are consulted to provide advice regarding diagnosis and management of patients with a range of conditions that are complex and challenging to assess [2–5]. One such condition is burns, commonly seen in many resource-scarce settings but challenging to diagnose by inexperienced physicians in all settings [6]. When expert diagnosis at bedside is not feasible, remote assessment may be a viable option. It is facilitated by modern forms of information communication technology (ICT) that allow for videoconferencing [7] or image-based consultation via smartphones [8]. In the case of burns, the point has been made that the latter can improve both triage and the referral process [9–12].

New systems of remote consultation to improve referral procedures have already been introduced in the USA and United Kingdom [13,14]. Remote diagnostic consultation among clinicians is also occurring through “direct to consumer” smartphone applications such as WhatsApp [9,15–17]. In both instances of changed forms of consultations, little is known as to how the experts themselves experience these changes and whether they influence the nature of their role and interactions with colleagues in need of their expertise at point of care [18,19].

The focus of this study is to understand how burns experts anticipate remote image-based consultation will affect their working methods and their relation with consulting clinicians. The following research questions are therefore considered: How do experts perceive the influence of a new image-based teleconsultation application on their interactions with remote colleagues? What changes in the practices around clinical diagnostic and advice do experts expect by the implementation of this new teleconsultation application?

Working as a medical specialist implies several roles and tasks that include interactions with colleagues. In the early 1990s, WHO launched the concept of the “five-star doctor” to expand the roles of general physicians with the attributes of: care provider, decision-maker, communicator, community leader and manager [20,21]. Research on roles of medical specialists has also been conducted in countries like Canada [22], US [23] and the UK [24]. These medical roles have been regarded as fairly stable over time in spite of comprehensive changes in how physicians work. To highlight the specific changes in expert practices when diagnosing remotely, the positioning theory was applied in this study [25]. The theory captures how individuals position themselves in interaction with others at the same time as they position others during specific, short situations such as remote consultations [26]. Positions are described as flexible and connected to a set of rights and duties ascribed to an individual in a specific situation [26]. Positions are seen as more dynamic and interchangeable than the more stable concept of roles and individuals can go in and out of positions during the same conversation [27]. When changes occur for example in this specific study, when new ways of working and new communications processes are introduced, positions can be challenged and new meaning is ascribed to the specific interaction [26]. We will use these theoretical considerations when analysing how medical experts described their expectations on changing consultation practices.

## Methods

### Setting

The study is set in the Western Cape in South Africa, a province with a population of 6.2 million [28], of which about 84% use the public healthcare services and the remaining 16% mainly use private providers [29]. Burn incidence in the province is high, and as in other settings, much related to poor and constrained living conditions [30–32]. There are published guidelines for referral protocols in the province that stipulate that minor burns should be treated at primary healthcare facilities and district hospitals while regional hospitals are expected to take care of both minor and moderate burns [33]. Any major and complex burns fulfilling the South African Burn Society’s criteria should be referred to either of the dedicated burns unit in the province (one for adults 13 years and older and the other for children 12 years and younger) [33]. It is also of note that the guidelines are accompanied by a ten-point plan to improve burn services in the province that includes the use of telemedicine [33].There is a high demand of service for burns care but resources are limited, both in specialised units (e.g. number of beds and specialists) and in primary and secondary facilities (e.g. clinicians able to assess burns) [33,34].

In line with the research questions mentioned above, expert clinicians were approached prior to the introduction and implementation of a smartphone application dedicated to acute burn diagnostic and treatment assistance (hereafter referred to as the “App”) for frontline clinicians (or point of care (POC) staff) [35]. The idea with the App is that POC staff can seek expert assistance provided that they fill in a set of standardised data on a given case (injury and patient, and upload pictures. At this stage, it is also possible to draw the burned areas on a figure appearing on the screen and a calculator thereafter estimates the total body surface area burnt. Once the request is sent (to a burns expert on duty), the software immediately supplies the POC staff with fluid requirements. All data are saved on a cloud server in a case format. The burns expert on call is then notified about the new case and uses the App or a web browser to review the case and gets back to the POC staff with a prompt reply regarding management advice. A more detailed description of the App can be found in a recent publication [35].

The original development of the App took place in the context of a research project involving South African and Swedish researchers [36]. After the prototype phase, the responsibility for the implementation of the App in the Western Cape Province was taken over by the South African partners who first introduced it to clinicians from a limited number of public health care facilities to improve its usability (e.g. by downloading the App on one’s own smartphone) and pilot test it. More recently, an attempt was made to integrate it in the referral system of the two burns unit of the Western Cape (under the name of the Vula App) [36]. The interviews with the specialists all took place prior to the implementation and independent of the South African partners.

### Design and participant selection

The study is based on qualitative, semi-structured interviews with medical experts. Purposive sampling was used to include a broad selection of experts who are future tele-experts for burns and included: all burns and emergency medicine experts planned to act as consultants in the App; senior level registrars from either speciality who had done one rotation at either of the burns units; an external tele-expert from another province to gain an outsider perspective selected on recommendation from the primary respondents. The participants were contacted via email to set up an appointment for the interview. Fifteen of the 18 invited accepted to be interviewed from March 5^th^ to April 17^th^. Their ages were from 27 years of age up to retirement age. Table 1 presents some basic characteristics of the participants with the level of detail adjusted to protect the privacy of the participants.

**Table 1:** Participants’ characteristics.

Specialty and experience	ID	Gender	Length of interview (in minutes)	Knowledge of the App prior to the interview
**Burns surgery (BS)** Accumulated experience in specialty (in years): 131 (range: 8–40)	BS. 1	M	53	Yes
	BS. 2	F	78	Yes
	BS. 3	M	76	Yes
	BS. 4	F	80	No
	BS. 5	M	56	No
	BS. 6	F	76	Yes
	BS. 7	F	71	No
**Emergency medicine (EM)** Accumulated experience in specialty (in years): 61 (range: 3–20)	EM. 1	M	44	Yes
	EM. 2	M	79	Yes
	EM. 3	F	53	Yes
	EM. 4	F	50	Yes
	EM. 5	M	90	Yes
	EM. 6	M	92	No
	EM. 7	M	95	No
	EM. 8	F	76	Yes

The investigation was initially informed by an information ecology framework that conceives the health care system as *“a system of people*, *practices*, *values*, *and technologies in a particular local environment”*[37]. The interview guide (see S1 File) contained broad open questions informed by a literature search on implementation and evaluation of telemedicine solutions with a special focus on user perspectives [14,38–40]. The interview started with questions about the participants’ current work situation, their experiences of diagnosing burns patients at bedside and remotely, and their interaction with POC staff, and continued with experience of using apps in their work and exploring perceptions on, and expectations of, the coming App. The interview guide was pilot-tested twice which resulted in a few changes although the overall structure of the guide remained intact.

### Data collection process and analysis

All interviews were conducted individually with two interviewers present. One interviewer had a clinical background but no prior involvement in the project while the other had a background in public health and had been involved in the early phases of the project (LB) in coordinating and conducting some of the field works for the project. These outsider and insider perspectives were used in eliciting current and future practices of the experts. The interviews took place at a time chosen by the participants and were conducted both during and after office hours. The interview locations included the participant’s or researcher’s workplace or home, or a café chosen by the respondent. The interviews lasted between 44 and 95 minutes (Table 1). They were recorded on two devices and were transcribed verbatim or intelligent verbatim. Both interviewers checked the content of the transcriptions independently.

Member checks [41] were conducted via telephone (with notes taken) for themes requiring further clarifications or explanations. Notes were also taken during visits to burns and emergency departments in the area. In addition, the second and third author held informal interviews or conversations with participants in Cape Town during the study period. Further details about the study design and data collection process have been published elsewhere [42].

Thematic analysis [43] was used to perform the data analysis and commenced with open coding of seven of the transcripts using both in vivo codes [43] and codes derived from the authors. The open coding was done throughout the whole transcripts, after which categories of types of interactions were created. For the following transcripts, the unit of analysis became those parts where the interviewees described the current situation of diagnosing burns, prior experiences of diagnostic apps and potential changes resulting from implementation of the App. When all transcripts had been coded, those first transcripts were revised again to check for consistency in the coding process. Thereafter, the codes were revised keeping in mind the two medical specialties of the interviewees to examine different patterns between them. By highlighting the social and clinical shifts described by the participants, the idea of using the positioning theory [26] emerged. It was used as a “tin-opener” [44] to capture the meaning of interactions with remote colleagues. The experts expressed diverse ways in understanding remote colleagues throughout the interviews and the positioning theory contributed to structuring the information and positions involved in these processes. The set of codes guided the formation of four positions.

### Ethical aspects

Ethical approval for the study was granted by the Stellenbosch University Health Research Ethics Committee (N15/01/008). Written informed consent from the participants was obtained prior to each interview.

## Results

**Fig 1:**
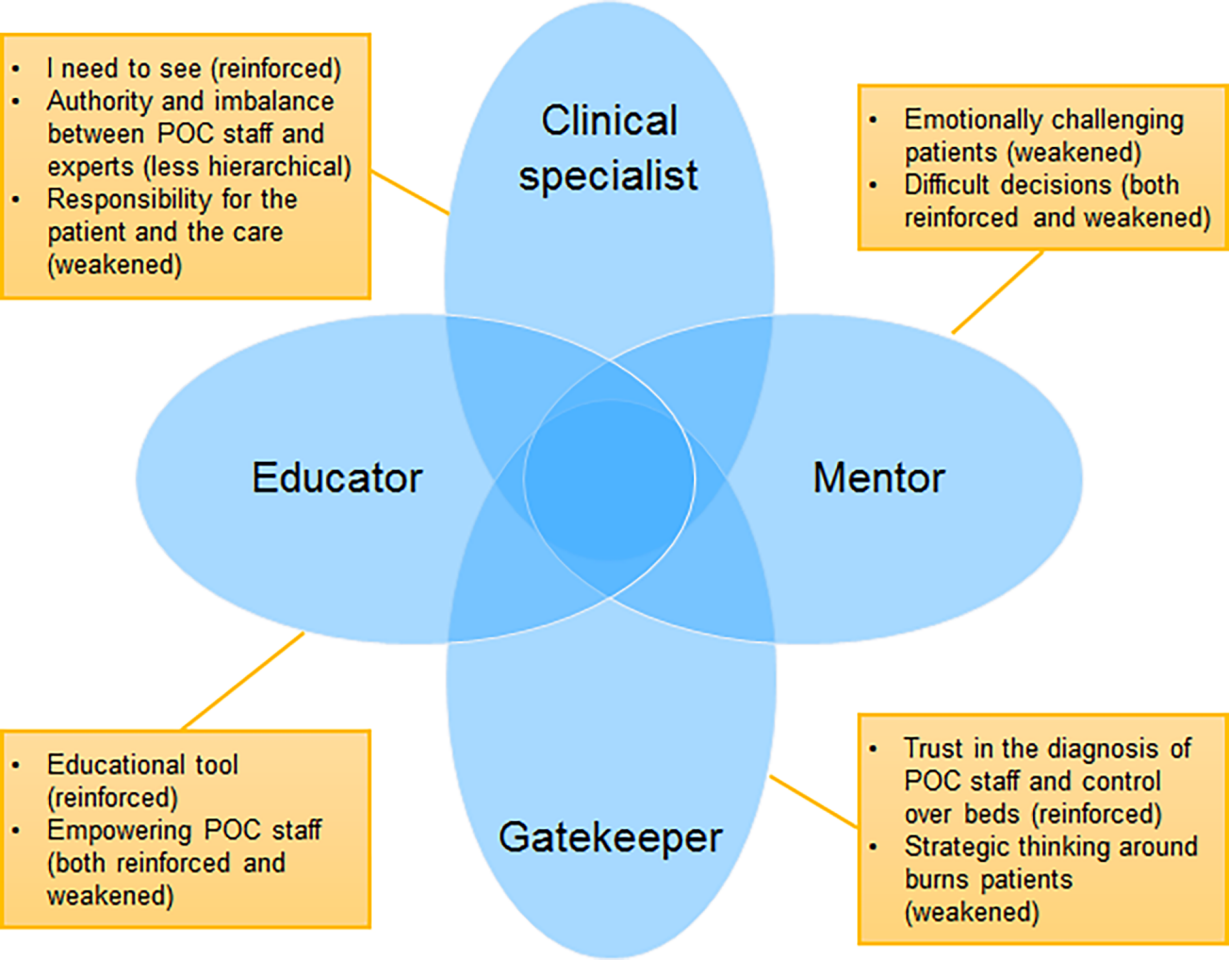
Four positions of experts with their related codes—Anticipated change by the App between parentheses.

Four main positions that the experts take on in their current practice were identified, of which two–clinical specialist and gatekeeper–are related to patient flow and the remaining two–mentor and educator–to the relation with POC staff (Fig 1). The experts move between these positions in a flexible manner in their general practice and more specifically during consultations with colleagues at POC. Each position is constructed by several codes. It is of note that at the time of the interviews, in addition to the conventional telephone consultations taking place, the interviewees were already engaged in consultation processes involving remote advice based on wound images, mostly via WhatsApp, and the positions were therefore already shifting compared with situations excluding image-based consultation.

### Clinical specialist

The clinical specialist position is the most prominent one in situations relating to diagnosing and managing patients. This position involves the clinical expertise and the authority and responsibility that are associated with that.

### “I need to see”

“It’s eyes on. I’m a surgeon, I need to see, even better, I need to feel, I need to be there, but if I can’t actually physically, I need to see because if you are not experiencing burns, what you are telling me, I can’t be sure that what I understand you to mean is actually what you mean.” (BS.7)

Most experts reported that images sent via WhatsApp often are used in remote consultations. This practice was user-driven, reportedly from both experts and POC staff and started a few years back. The experts consider that images facilitate diagnosis and improve the reliability and accuracy of the information provided to them. They perceive staff at POC as insecure when it comes to diagnosing a burn and the use of images compensates for the difficulties and provides a structure in the communication with POC staff.

The experts attached high importance to the circumstances of how the injury occurred when diagnosing. Both pre- and post-initiation of WhatsApp, this information was often transferred verbally over the phone while the experts used the opportunity to interpret the reliability of the information of the referring staff. The experts therefore envision the App as a complement but not as a substitute to verbal communication.

In that respect, the App is a formalisation of the current practices and the participants anticipate that the structured frame it provides will ensure the right clinical information is communicated. Some of the experts raised a concern about a potential “in-built delay” of communicating through an App, with login, filling in text fields etc., compared to the directness of a phone call.

### Authority and the imbalance in power between POC staff and experts

Authority and imbalance in power between POC staff and experts (Fig 1) is a complex topic that experts were concerned about. With or without images in the consultation process, the experts depict a culture where asking for help is uncommon, where POC staff are hesitant to request expert advice, and experts could be hard to get in touch with. They describe experiences where POC staff avoid setting a diagnosis of the burns, as one of the experts stated:

“They’re usually quite good at telling me the story, but they can’t diagnose the burn. So I’m like: “How big is the burn and how deep is it?”, so they’re very good at the story and I think that’s linked to their insecurity and their lack of training to do the evaluation. They don’t tell me that [depth and size] because they know they might not be right.”(BS.6)

The experts perceive that power relations might be altered when the App is in place by making experts more accessible when POC staff know that there is an agreed-upon system put in place to facilitate the contact with experts. In addition, the anonymity that the App is expected to bring is suggested to make it less intimidating for POC staff when the experts are not able to reflect on who is asking for advice.

#### Responsibility for the patient and the care

Being a clinical specialist comes with a sense of responsibility for the patient and the care. Although the legal responsibility of the patient remains with the POC facility, the experts consider themselves as partly responsible for the care of the patient when providing remote advice. They perceive passing on responsibility from POC staff to experts as one of the reasons why POC staff contact them. The experts are concerned that the App will dilute the power of the expert:

“What do you say when you’re trying to write that down [patient consulted through the App] in your notes, as opposed to “Doctor [Smith] said that he thinks I should do this”, which is great because medico-legally I believe that that means I’m safe. It doesn’t necessarily …but someone senior has said that, now I’m writing “the burns App”, or “the burns expert said”–it might not be as powerful.” (EM.8)

POC staff were considered responsible for providing the experts with accurate information, verbally and visually by sending photographs adequately representing the burns:

“You advise him it’s okay: “I don’t think you need to intubate”. But he’s not showing you what you want to see and if you can see that, once again, the guy that’s on that side, must realise he is taking responsibility for what he is sending through to you and what he is giving to you and you cannot take his responsibility” (BS.2)

The visualization via smartphones is perceived to improve the quality of the advice from remote experts, approaching the quality of a diagnosis made bedside. This has blurred the lines of responsibility and the legal grey areas are expected to remain.

#### Gatekeeper

The experts are gatekeepers to beds at different levels of care, some at the burns unit and others at regional or district hospitals. Similar to the clinical specialist, the gatekeeper position is already shifting with the practice of using images in the referral process. The two positions overlap since an accurate diagnosis supports getting the right patient to the right place.

#### Trust in the diagnosis of POC staff and control over beds

The experts express high satisfaction with including images in the referral process. They explain that images help to ensure that POC staff give them correct information which in turn increases their trust in the transferred information. Images are specifically useful for borderline cases when the necessity for referral is uncertain and images are perceived to improve the efficacy of the referral and speed up the referral process:

“We’ll use WhatsApp, saying “This is what I’ve got, what do you think?” and we’ll make a decision on transfer from those pictures. And they can also e-mail us; then ultimately we use a formal e-mail reference. But for that initial decision it’s all made on cell phone images.” (BS.4)

From the experts’ perspective, there is a hope to address data security by the introduction of the App, making it safer to send images between healthcare providers:

“What I would like to see is more formalisation like this App, as opposed to people using WhatsApp, which is where we are coming up with, people are just firing pretty much confidential images around at random.” (EM.1)

The experts suggest that the App could streamline the process assuring that the right clinical information is transferred together with the images:

“To have an App, it just makes it more structured. The App asks you the right questions, so you give the right answers…..That’s really helpful because like I said, people don’t always know what’s important” (BS.7)

The experts highlighted existing problems in the referral process making it hard to adhere to South African Burn Society’s referral criteria. They envision the App as only partially improving that process by selecting the patients in most need of referral but not addressing the problem of few beds. The experts fear that the use of the App could be seen as irrelevant by POC providers if advice from the experts cannot be followed up due to lack of a referral bed.

#### Strategic thinking around burns patients–it’s not just the burn

For a gatekeeper, decisions on referral are not only based on clinical conditions but also on strategic thinking including contextual factors. While the experts describe some clinical criteria for referral to be universal, like inhalation burns, contextual factors related to the capacity of the remote health facility and the circumstances of the patient and his/her family, are harder to communicate succinctly. This is in particular a concern when staff at POC are insecure, not used to managing burns patients or do not feel sufficiently equipped to take care of the patient. In remote Western Cape, where patients often come from difficult living conditions and have long distances to travel for specialised care, experts underline the importance of taking the patient’s social situation into account for decisions around management and disposition:

“Just looking at pictures won’t necessarily dictate that that patient needs to be transferred itself. It’s the setting where the transfer is coming from that would influence one’s decision” (BS.5)

This kind of information is currently communicated verbally. The experts expressed some concern that contextual information could be missed when using the App.

### Mentor

The experts perceive providing moral support to POC staff and more junior doctors to be an important task in their day-to-day work.

#### Emotionally challenging patients

Burn patients are often in terrible pain and with several body functions in danger. The feeling among the experts is that these patients are viewed by POC staff as emotionally challenging and that support from more experienced colleagues to make the best decision on burn managements is essential:

“People who are not used to working with it, they kind of cringe at the painfulness of it, they’re scared of the resuscitation, they’re scared of the airways. That is something that makes most doctors fearful. Definitely.”(BS.4)

In this regard, experts also often move back and forward between the mentor and the gatekeeper position, considering insecure POC staff as a criteria for referring the patients, both for the sake of patients and worried clinicians. This kind of support is also seen as very important when something goes wrong and referring staff blame themselves. This position has not changed much following the introduction of image-based communication and it is not expected to change much either with the App since verbal communication is considered essential, more than in any of the other positions.

#### Difficult decisions on severe burns

The most extreme decisions where support from more experienced colleagues is indispensable are those cases where patient survival is uncertain. In addition, scarce resources and limited number of intensive care unit beds have to be taken into account by the referring expert:

“So it’s basically like a crisis situation you know, the less disaster situation we have to decide this one will make it, this one won’t make it, just because of our resource limitations and our high demand for services” (BS.3)

When experts express moral support, some of them look back on their own time as junior doctors and recall the feeling of ending up responsible for making decisions around severely burnt patients without having the experience and knowledge they nowadays possess:

“I think for the juniors to know, they feel like someone is in their corner. Because it’s really hard when you’re working in a more peripheral hospital. You’re community service, you’ve just finished your internship and suddenly you’re the senior on the floor.” (BS.7)

The experts describe these decisions as extremely challenging and they see themselves as a person to provide support, “someone out there” that they never had. In these situations, the mentor position covers the responsibility of taking the ultimate decisions but also to support POC staff emotionally and guide them in how to proceed with palliative care, documentation and informing and handling patients’ families and close ones:

“Being overwhelmed by this really bad burn and knowing that it’s wrong for them to take up a bed in some way and having to make that decision, particularly for a junior provider to say well, I’m not going to do anything for this patient, I’m going to leave them here to die, or I’m going to let their family come in and then I’m going to intubate them and its …that actually needs quite a lot of support.” (EM.8)

Images are perceived as useful in this situation to support the setting of an accurate diagnosis. They enable the experts to alternate more effectively between the mentor and the clinical specialist position both in the process of diagnosing and in taking responsibility for decisions:

“I will usually ask them to send me a photo so I can just see what they are seeing, to just see if they are giving me an accurate description of the burn depth, because the worst thing is that you don’t want to make an error when it comes to withdrawal of therapy…” (BS.3)

It is suggested that diagnosis of severe cases could be made even more accurate through the App and that the App could include a few basic principles on how to proceed with palliative care. However, also around these difficult decisions, the App is not regarded as a substitute for the emotional support by the experts and phone calls would continue to be essential:

“The advice, moral support, again, I’m not sure it would make a huge difference to that because the big burns, the moral support that you give for the big burns, I don’t think it would change. I think they’d be phoning those anyway.” (BS.7)

The position is expected to remain relatively similar even when the App is implemented.

### Educator

Education of POC staff is seen by experts as a vital part of their work. They describe several benefits of improving training of POC staff, expanding clinical skills, making burns care a more attractive option for specialisation and in the longer perspective empowering staff at POC.

#### Educational tool

Prior to the use of images, the experts’ educational efforts were primarily directed towards staff within a unit and the experts had more of a controlling approach towards remotely located physicians. The position grew in scope once images started to be used in the consultation process:

“And it’s also a nice teaching tool [WhatsApp], because now you will explain to that person why you think it’s actually a 12% and not a 40% [burn] and in that process they also …you know, you do a little bit of teaching.” (BS.4)

The relationship building feature provided with WhatsApp is also seen as a beneficial addition. The structured approach of the App is seen to provide an additional advantage as an educational tool:

“So I think the App will offer a more structured approach and the doctor also will learn about the information he needs to gain. Because the App will guide you: How was the patient burnt? How old is the patient? The structured approach….helps the junior doctor to form processes.” (BS.6)

Participants suggested that the App could complement the experts in their efforts to educate POC staff where the repetitive way of filling in and going through information provided by experts is assumed to facilitate learning.

#### “Empowering POC doctors”

The experts propose that these new technological solutions could contribute to bring more attention to burns care in general, raising the status of burns care and making it more meaningful:

“Burns has not been regarded by a lot of junior staff as glamorous or as an attractive area within …it’s sort of regarded as somewhat of a burden to people at the primary level. I think coming into the 21st century using a sophisticated app that is easy to use and effective will kind of make things a little bit more …maybe glamorous isn’t the right word, but more modern.” (BS.5)

The experts speculate that the hesitancy among POC staff towards burns patients is connected to a lack of training. Empowering POC staff is one of the strategies to make them feel more secure and in control of difficult situations and the experts move between the educator and the mentor position when doing this:

“When you get it right, it’s extremely rewarding and again, it’s about empowering people and education to feel equipped. So I’m involved in a lot of burns teaching and training and it’s such a huge difference to see somebody that you know really had an aversion to burns, as soon as you equip them, it’s really not so bad” (BS.6)

However, the experts also express the benefits of a pleasant voice in encouraging and empowering POC staff, something that the App in itself would lack.

## Discussion

The difficulty of diagnosing burns makes expert advice an often crucial support for diagnosis. In this study, this is demonstrated in the way that experts take on different positions in relation to POC staff, some of which are more prominent here than in previous studies. Furthermore, the experts are already facing changes in their practice due to user-driven changes introduced by open access ICT tools. In this sense, the concept is already in place and this overlap between the App and WhatsApp provides the experts with something to relate to when discussing the App. This study is one of the few seeking to explore the perceptions of experts of an image-based teleconsultation system: they are often initiators of such systems but seldom approached for their views on how the systems could influence the consultation process and their relations with other healthcare professionals. All four positions are expected to remain during diagnostic practices with the App in place.

The experts’ positions had at the time of the interviews gone through considerable changes, demonstrating the experts’ openness to adjust in a rapidly changing context of burn diagnostic practices. In this setting, healthcare professionals are not waiting for a system to be built but are actively trying to improve practices involved in remote diagnosis by using informal channels, exemplified by WhatsApp. WhatsApp is gaining ground in the medical field [9,16,45] and is a more general application than the App presented here. The App is specifically designed for the purpose of burns consultation and tailored for the expertise of the experts. Several advantages like reduced consultation time [46], decreased hierarchy [16,47] and making senior physicians more accessible and involved in decisions [16,47,48] have been described by using WhatsApp, along with disadvantages such as incompatibility with medical record keeping [49], problems of data security, patient consent, confidentiality and privacy [17,45,50,51]. The end-to-end encryption of WhatsApp since April 2016 has improved security of data during transmission [45], although issues around storage of data persist [9,52].

The mentor is a novel position in relation to previous research on medical roles [20–23,53]. Experts emphasised this empathetic aspect of expert advice. The reason for this could be related to the specifics of being a middle-income country where experts are present along with POC staff that are given considerable responsibility early on in their careers, all in a pressured healthcare system. In addition, the emergency care environment where decisions have to be taken rapidly and where POC staff see a broad variety of diseases and conditions could also contribute.

A key characteristic of a healthy information ecology according to the information ecology framework is the coevolution of social and technical aspects, constantly evolving and adjusting towards each other and where balance is “*found in motion and not in stillness*”[37]. Using positions in the context of medical competencies instead of the more stable concept of roles captures the dynamic character of diagnostic consultations where experts can be in more than one position simultaneously and the experts go in and out of positions in one and the same consultation. This overlap is especially large between the clinical specialist, gatekeeper and the mentor. The experts shift for example between the clinical specialist and the gatekeeper when making an accurate diagnosis on which to base the referral decision. Similarly, the clinical specialist and the gatekeeper overlap with the mentor during difficult decision making processes where patient survival is uncertain, both to take over responsibility distantly or, if needed, physically by transferring the patient.

The need for verbal communication during diagnosis may persist and is mentioned in several of the positions. Its importance is particularly emphasised for the mentor position. This position is the least likely to change following the implementation of the App since the App in itself does not include expressions of empathy to POC staff and is not expected to substitute for that emotional support. The results suggest that the App could provide several benefits to the other three positions. The experts expressed confidence in the educational potential of the App and the educator position is expected to be reinforced with the App in place. Indeed, educational benefits [54,55], as well as feelings of empowerment among healthcare workers [56] from using mobile apps have been demonstrated in studies from other areas. For the clinical specialist and the gatekeeper, it has been suggested that image-based communication via smartphones will streamline the remote diagnostic process by speeding up communication [10,49,57], and by improving both triage and referrals [9–12,57,58]. However, these technical solutions might be at the expense of the interpersonal sides of the communication and there could be some information that the App has more difficulties to provide even for the clinical specialist and the gatekeeper position.

Turning to the robustness and the trustworthiness of the results, a few potential sources of bias should be mentioned. One is that some of the interviewees had prior knowledge of the App from having heard about it or seen the prototype prior to the interview. As all of them had familiarity with some form of remote consultation, it is difficult to assess how big of a bias the prior knowledge actually is. One of the two interviewers that conducted the interviews was known to some of the interviewees. Whether this has influenced their expressed expectations of the App is uncertain. In any case, all interviewees came with suggestions for improvements.

Further, as the targeted group of potential participants is small, privacy could have been a concern among the interviewees in spite of the efforts of the research group to preserve anonymity. However, the response rate was high and the interviewees were committed and willing to reflect and share positive as well as negative examples of their current practice and expectations of the App both in the interview and the member checks.

Finally, the results are likely to be transferable [59] to similar areas characterised by large burden of burn injuries, long distances between facilities, and scarce specialised resources. The study sample is limited to the expert’s perspective, which was the focus of the study. Nonetheless, the perspective of POC providers could have enriched the analysis further by providing another perspective on the roles and interaction with experts. The selection of participants covers a broad variety of perspectives of different specialties, years of experience and ages of the participants which make the results more robust. Furthermore, in this context characterised by a high number of burns patients, the experts are consulted regularly in their day-to-day life which is why the topic of the interview is well-known to them. The information power [59] of the sample could be considered strong since the aim of the study is well-focused, the sample of participants is purposive and the participation rate is high, the study is informed by theory and the answers from the participants were rich and detailed.

## Conclusions

Experts described four shifting positions in current remote consultations via phone or WhatsApp. There was already some flexibility in the positions prior to project implementation exemplified by the use of WhatsApp. A new position, the mentor, was identified which could reflect the high pressure on healthcare professionals in this setting. All positions are expected to be changed following introduction of the new App, although spoken communication might continue to be essential for some situations. Experts’ reflections on remote consultations highlight the embedded social and cultural dimensions of implementing new technology.

## Supporting information

S1 File Interview guide. (DOCX)
